# High-Fat Diet Propelled AOM/DSS-Induced Colitis-Associated Colon Cancer Alleviated by Administration of *Aster glehni* via STAT3 Signaling Pathway

**DOI:** 10.3390/biology9020024

**Published:** 2020-02-02

**Authors:** Bo-Ram Jin, Kyung-Sook Chung, Minho Lee, Hyo-Jin An

**Affiliations:** 1 Department of Pharmacology, College of Korean Medicine, Sangji University, 83 Sangjidae-gil, Wonju-si, Gangwon-do 26339, Korea; wlsqh92@gmail.com; 2 Department of Pharmaceutical Biochemistry, College of Pharmacy, Kyung Hee University, Seoul 130-701, Korea; adella76@hanmail.net; 3 Catholic Precision Medicine Research Center, College of Medicine, The Catholic University of Korea, 222, Banpodaero, Seocho-gu, Seoul 06591, Korea

**Keywords:** high-fat diet, colitis-associated colon cancer, inflammation, *Aster glehni*

## Abstract

Many epidemiological observational studies suggest that a high-fat diet (HFD) accelerates the risk of colorectal cancer (CRC). Inflammation can play a key role in the relationship between colon cancer and HFD. Although reported by several studies, controlled experimental studies have not explored this relationship. We established an HFD-fed colitis-associated colon cancer (CAC) mice model and evaluated the anti-tumorigenic effects of AG on HFD-propelled CAC along with its mechanism of action. Previously, we found that *Aster glehni* (AG) exerts chemopreventive effects on azoxymethane (AOM)/dextran sulfate sodium (DSS)-induced CAC in a mice model, and has anti-adipogenic effects in a HFD-induced obesity mice model. In the HFD-propelled CAC mice model, AG significantly reduced cancer-related death, prevented body weight loss, and alleviated splenic enlargement. Additionally, AG prevented colon shortening and reduced the number of colorectal polyps. Histological studies demonstrated the up-regulation of inflammation, hyperplasia, and neoplasia in HFD-propelled CAC mice, whereas AG suppressed colonic disease progression and tumorigenesis. Furthermore, AG significantly inhibited the signal transducer and activator of transcription 3 (STAT3) signaling pathway and attenuated the protein expression of the STAT3 target gene, which mediates transcription factor-dependent tumor cell proliferation. These results indicate that AG abrogates inflammation-induced tumor progression in HFD-propelled CAC mice by inhibiting STAT3 activation.

## 1. Introduction

Colorectal cancer (CRC) is the third common malignant tumor and one of the leading causes of cancer-related death among both men and women worldwide [1]. The major cause of CRC is thought to be environmental-related lifestyle risk factors, with <20% of cases associated with hereditary factors [2]. It is worth noting that CRC development can be initiated by inflammatory signals. Colitis associated cancer (CAC) is a type of CRC which is preceded by inflammatory bowel disease (IBD), such as ulcerative colitis and Crohn’s disease. The relative risk of CRC in patients with IBD has been estimated between four-and 20-fold and the actual risk of CAC directly correlates with the severity and longevity of IBD [3]. The usage of anti-inflammatory treatment, including 5-aminosalicylic acid (5-ASA) and aspirin, is associated with protection against CRC, a finding that to a large extent mirrors that of inflammation as a risk factor for CRC [4].

The association between obesity and CRC incidence has been evaluated in several epidemiological studies, and it has been suggested that the risk and incidence of CRC increase with obesity [5]. Previous studies have reported that high-fat diet (HFD)-fed mice increases intestinal barrier dysfunction and promotes the development of CRC, indicating inflammation due to HFD plays a role in the increased risk of CRC [6,7]. It is believed that inflammation plays a role in CRC progression and HFD prompts inflammation. Prolonged HFD induces low-grade chronic intestinal inflammation in mice, and HFD is a risk factor for the development of IBD via impairment of the intestinal barrier function [8]. Although there is evidence to indicate that excess fat intake accelerates the risk of CAC, there are limited in vivo models to understand the molecular signals. Here, we established experimental models to address the effects of high-fat intake on inflammation-associated colon cancer development and progression, and provide mechanisms involved in such a relationship.

Signal transducer and transcription activator 3 (Stat3), which is a point of convergence for many oncogenic pathways, has emerged as a critical regulator for tumor-associated inflammation [9]. STAT3 is a latent cytoplasmic transcription factor that becomes activated by phosphorylation at tyrosine residue 705 (Tyr705). Phosphorylated STAT3 (pSTAT3) proteins dimerize and translocate to the nucleus, where they transcribe the expression of various genes involved in cell cycle progression, proliferation, migration, invasion, and survival [10]. Activation of STAT3 induces the expression of anti-apoptotic gene Bcl-2 that also correlates with the tumor invasion, metastasis, and worse prognosis in CAC [11]. Enhanced pSTAT3 expression was detected in epithelial cells of CAC patients, and several studies have documented that STAT3 serves as a target for CAC immunotherapies [12,13]. Although the exact role of the STAT3 pathway has not yet been elucidated in HFD-accelerated CRC, previous studies reported that HFD exacerbated the extent of cancer progression via STAT3 signaling in various diseases, suggesting that the STAT3 signaling pathway is one of the underlying molecular mechanisms involved in HFD-propelled tumorigenesis [14,15].

*Aster glehni* Franchet et Sckmidt (AG), a traditional edible herb from the Republic of Korea, has been used to treat diabetes, hypercholesterolemia, and coronary artery disease [16]. Previously, we reported that AG has anti-inflammatory effects in the dextran sulfate sodium (DSS)-induced colitis mice model, and has chemopreventive effects in AOM/DSS-induced CAC mice model [17,18]. In addition, we observed that AG has anti-adipogenic effects in the HFD-induced obesity mice model. It is possible, therefore, that the administration of AG may result in therapeutic effects against the development of HFD-propelled CAC. In the present study, we aimed to evaluate the effects of AG on a mice model of HFD-propelled colitis-associated tumorigenesis and then investigate the molecular mechanism responsible for the therapeutic effects of AG.

## 2. Materials and Methods

### 2.1. Chemicals and Reagents

Dextran sulfate sodium (DSS) was purchased from MP Biomedicals. The primary antibodies for STAT3 (catalog number sc-482), Cdk4 (sc-23896), Bcl-2 (sc-7382), and β-actin (sc-47778) antibodies were purchased from Santa Cruz Biotechnology. p-STAT3 Tyr705 (#9145) was acquired from Cell Signalling. Peroxidase-conjugated secondary antibodies were purchased from Jackson ImmunoResearch, Inc. Azoxymethane (AOM), 5-aminosalicylic acid (5-ASA), camptothecin (CPT), H&E, and all other chemicals were purchased from Sigma-Aldrich Co.

### 2.2. Experimental Animals and Diet

Male Balb/c mice (*n* = 60; 8 weeks old; 24–26 g; Daehan Biolink) were housed 10 per cage in the animal room with 12 h dark/light cycles and constant temperature (temperature, 20 ± 5 °C; humidity, 40–60%) with free access to food and tap water. All animal experiments were conducted following the university guidelines and were approved by the Animal Care Committee of Sangji University (Reg. No. 2016-11). Except for animals in the normal group, all animals were fed on HFD. The normal diet consisted of a standard laboratory chow (NIH-41 open formula diet; Zeigler Bros., Inc., Gardners, PA, USA) with 5% fat, whereas the HFD contained 45% fat (D12451 open formula diet; Research Diets, Inc., New Brunswick, NJ, USA).

### 2.3. Preparation and Standardization of the Extract of AG

*Aster glehni* (AG) extracts were prepared as described previously [18,19]. The dried material was refluxed with 70% EtOH for 6 h at 60 °C. The extract was filtered (Whatman Qualitative Filter Paper no. 4, 20–25 µm, GE Healthcare Life Sciences, Seoul, Korea), concentrated under reduced pressure, and then freeze-dried (−50 °C, under a pressure between 20 and 30 Pa) to obtain a solid extract powder (73 g).

### 2.4. AOM/DSS-Induced CAC Model and Treatment

**Figure 1:**
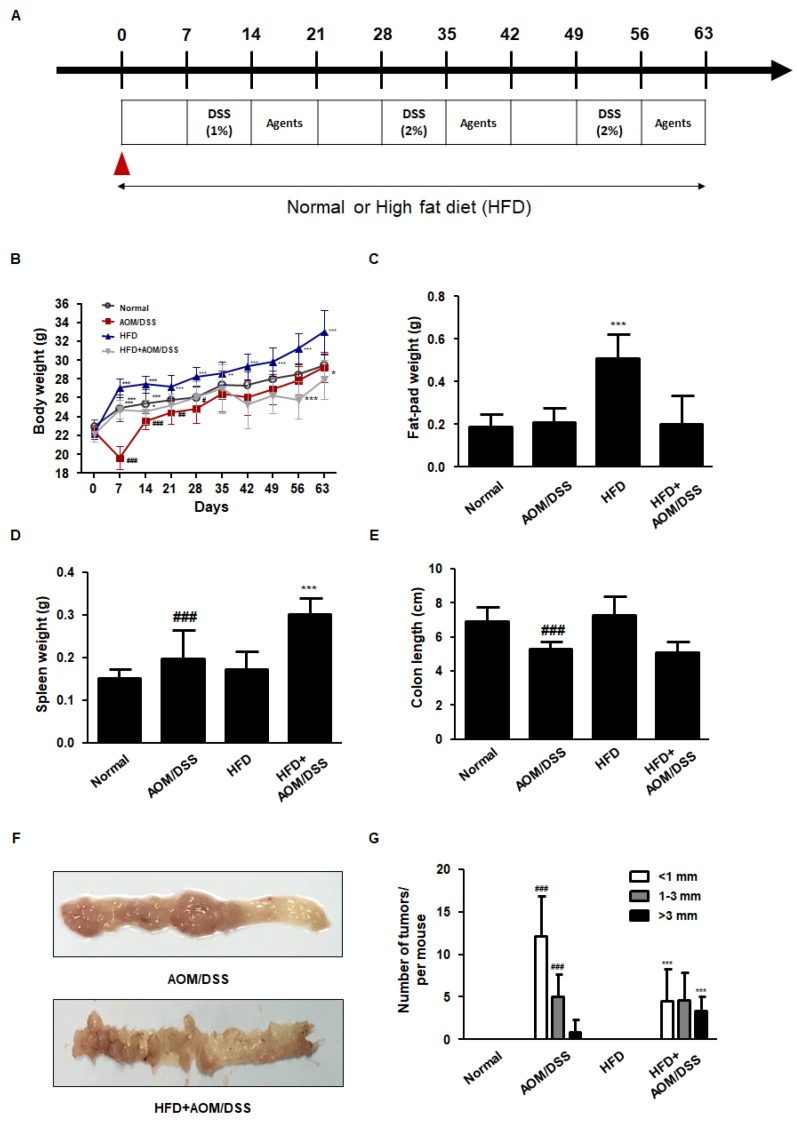
Effects of HFD on the pathological symptoms in the AOM/DSS-induced CAC mice model. (**A**) Scheme of the AOM/DSS-induced CAC mice fed on normal diet or HFD. (**B**–**G**) The animals were randomly divided into four groups: normal group, AOM/DSS group (AOM/DSS-induced CAC mice), HFD group (mice fed HFD), and HFD+AOM/DSS group (AOM/DSS-induced CAC mice fed HFD). (**B**) Body weight, (**C**) fat-pad weight, (**D**) splenic weight, and (**E**) colon length were evaluated in each mouse. (**F**) Representative photograph of the colon tissues from the AOM/DSS and HFD+ AOM/DSS groups is shown. (**G**) The number of tumors in each group was measured. The values are the mean ± SD (*n* = 10); ^###^ *p* < 0.001 vs. normal group; * *p* < 0.05, *** *p* < 0.001 vs. AOM/DSS group; significant differences between the treated groups were determined by ANOVA and Dunnett’s post-hoc test.

The AOM/DSS method has been established to induce inflammation-induced CRC [17,20]. To establish the CAC model, we injected each animal intraperitoneally with 12.5 mg/kg of AOM dissolved in PBS. After 7 days, the animals were provided with drinking water containing 1% DSS for 7 days, followed by drinking water for 14 days, and exposed to two more 2% (*w*/*v*) DSS treatment cycles. On day 14, we administered ASA (75 mg/kg daily, p.o.), CPT (0.5 mg/kg daily, i.p.), or AG (25 and 50 mg/kg daily, p.o.) to the mice daily for a week after suspending DSS treatment (Figure 1A). For the positive controls, we used 5-ASA, a drug used to treat IBD, and CPT, an anticancer drug that inhibits the type I DNA topoisomerase. The mice were weighed weekly and sacrificed on day 63. 

### 2.5. Histopathological Examination

The segregated colon samples were fixed instantly with 10% (*v*/*v*) formalin and embedded. The paraffin-embedded samples were sectioned to 5 μm thickness and stained with H&E and periodic acid-Schiff (PAS). All processes were followed as described previously [21]. In terms of histopathological analysis, the description of the parameters used to score the degree of hyperplasia and inflammation are presented in Table 1 and Table 2, respectively. 

### 2.6. Immunohistochemistry (IHC)

IHC was performed with the formalin-fixed, paraffin-embedded sample. The paraffin blocks were cut into 5-μm-thick sections, mounted onto poly-L-lysine-coated slides, and dried. After the dried slides were de-paraffinized, antigen retrieval was performed using an automated antigen retrieval machine for 20 min with cell conditions of ethylenediaminetetraacetic acid (pH 9.0). The non-specific binding to the sections was blocked by incubating for 1 h with 15–20% (*v*/*v*) normal goat serum (Gibco Life Technologies) prior to incubation with the appropriate primary antibodies for 2 h at room temperature or overnight at 4 °C. The secondary rabbit antibodies were used to detect the primary antibodies, followed by streptavidin-tagged horseradish peroxidase (Ventana Medical Systems). Diaminobenzidine (DAB; Sigma-Aldrich, St. Louis, MO, USA) was used to induce signaling, and Bluing Reagent (Ventana Medical Systems) was used as a counterstain. The IHC slides were visualized under an optical microscope (Leica) and rendered using Leica software. The IHC staining of the antibodies to p-STAT3 (Tyr705) was examined.

### 2.7. Western Blot Analysis

The proteins were extracted from the mice colon tissues utilizing the protein lysis buffer (Pro-prep, Intron). The quantified samples were fractioned on an 8–12% (*w*/*v*) gradient SDS gel, transferred to PVDF membrane, and probed with specific primary antibodies in T-TBS (2.5% (*w*/*v*) skim milk) at 4 °C. The peroxidase-conjugated secondary antibodies (Jackson ImmunoResearch) were incubated at 25 °C. The bands were visualized using the ECL solution (Ab signal) and displayed on an X-ray film (Agfa).

### 2.8. Statistical Analyses

All experiments were performed in triplicate, and the data are expressed as the mean ± standard deviation (SD). Analysis of variance (ANOVA) and Dunnett’s post-hoc test were performed. A *p*-value of ˂0.05 was considered statistically significant. All statistical analyses were performed using GraphPad Prism 5.

## 3. Results

### 3.1. HFD Accelerated the Development of AOM/DSS-Induced CAC Mice Model

In this study, we established an HFD-fed CAC mice model to examine the effect of HFD on the pathogenesis of AOM/DSS-induced CAC mice model. As shown in Figure 1B,C, HFD+AOM/DSS showed a loss of body weight with no effect on fat-pad accumulation. In addition, HFD+AOM/DSS increased the enlargement of the spleen as compared to that in the AOM/DSS group, alluding that HFD increased susceptibility to the inflammatory environment. Meanwhile, HFD did not have any significant impact on the colon length as compared to that in the AOM/DSS group. As shown in Figure 1F,G, macroscopic tumors were observed in the mice on AOM/DSS treatment, and HFD+ AOM/DSS group mice showed a significantly high level of multiplicity as compared to those in the AOM/DSS group. 

### 3.2. HFD Propelled Tumorigenesis and Tumor Progression in AOM/DSS-Induced CAC Mice Model

**Figure 2:**
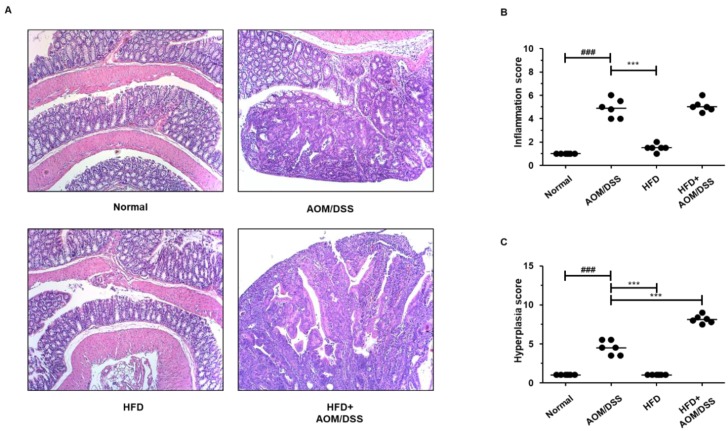
Effects of HFD on the histological analysis in the AOM/DSS-induced CAC mice model. (**A**) A representative portion of the colon tissues was stained by H&E (original magnification, 100×). (**B**) The inflammation score and (**C**) hyperplasia score were estimated. The values are the mean ± SD (*n* = 10); ^###^ *p* < 0.001 vs. normal group; *** *p* < 0.001 vs. the AOM/DSS group; significant differences between the treated groups were determined by ANOVA and Dunnett’s post-hoc test.

Next, we examined the histomorphological evaluation of intestinal inflammation, hyperplasia, and tumorigenicity using hematoxylin and eosin (H&E) staining. The HFD+AOM/DSS group showed severe morphological progression as compared to that in the AOM/DSS group (Figure 2 and Table 3). The inflammation score in the AOM/DSS and HFD+AOM/DSS was similar, whereas there was a 1.8-fold increase in the hyperplasia score in the HFD+AOM/DSS group as compared to that in the AOM/DSS group. In the cancerous area, the number of the tumor increased significantly to 1.9-fold in the HFD-fed AOM/DSS group. Furthermore, HFD+AOM/DSS mice revealed notable progress in high-grade macroadenoma and advanced adenocarcinoma compared to that in the AOM/DSS group. Collectively, these findings suggest that HFD increased the susceptibility to AOM/DSS and accelerated the development of inflammation-induced CRC. 

### 3.3. AG Ameliorated Survival and Pathological Symptoms in HFD-Propelled CAC Mice Model

**Figure 3:**
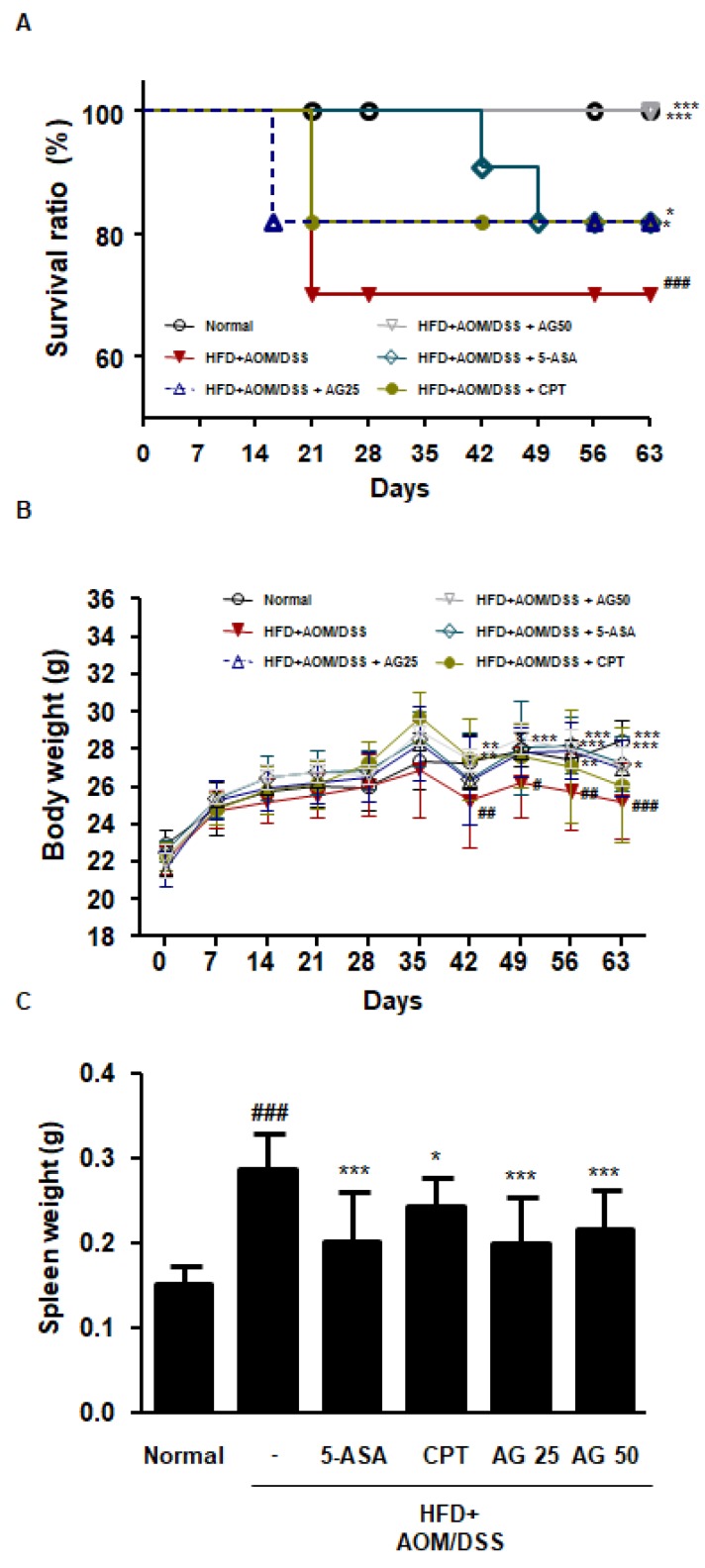
Effects of AG on the pathological symptoms in the HFD-propelled CAC mice model. The animals were randomly divided into six groups: normal group, HFD+AOM/DSS group (AOM/DSS-induced CAC mice fed HFD), 5-ASA group (CAC-induced mice fed on a HFD which was administered with 5-ASA 75 mg/kg daily, p.o.), CPT group (CAC-induced mice fed on a HFD which was administered with CPT 0.5 mg/kg daily, i.p.), and AG 25 and 50 group (CAC-induced mice fed on a HFD which was administered with AG 25 and 50 mg/kg daily, p.o.). (**A**) The survival ratio, (**B**) body weight, and (**C**) splenic weight were measured in each mouse. The values are the mean ± SD (*n* = 10); ^##^ *p* < 0.01, ^###^ *p* < 0.001 vs. normal group; * *p* < 0.05, ** *p* < 0.01, *** *p* < 0.001 vs. the HFD+AOM/DSS group; significant differences between the treated groups were determined by ANOVA and Dunnett’s post-hoc test.

Our previous studies have shown that AG reduced the tumor number and size in AOM/DSS-induced CAC mice model. In the present study, we investigated whether AG has chemopreventive effects on the HFD-propelled CAC mice model. According to the Kaplan–Meier survival curves, the survival ratio of the HFD+AOM/DSS group significantly decreased in contrast to that of the normal group. However, the administration of 5-ASA, CPT, and AG alleviated this effect (Figure 3A). As observed in earlier studies, exposure to AOM and DSS caused a remarkable reduction in body weight and an increase in splenic weight, whereas the administration of 5-ASA, CPT, and AG restored body weight and reduced the splenic weight (Figure 3C). 

### 3.4. AG Suppressed the Development of Colonic Neoplasms in HFD-Propelled CAC Mice Model

**Figure 4:**
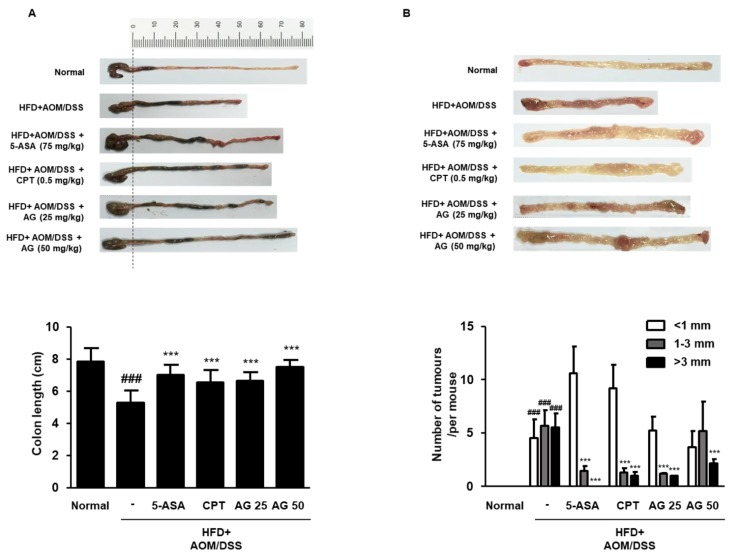
Effects of AG on the colonic tissues in the HFD-propelled CAC mice model. (**A**,**B**) The colons were obtained after 63 days of AOM treatment and the representative photographs of colon tissues from each group are provided. The length of the colon of each animal was measured between the caecum and proximal rectum. (**A**) The colon length and (**B**) the number of macroscopic tumors were measured. The values are the mean ± SD (*n* = 10); ^###^ *p* < 0.001 vs. normal group; *** *p* < 0.001 vs. the HFD+AOM/DSS group; significant differences between the treated groups were determined by ANOVA and Dunnett’s post-hoc test.

The shortening of colon length is the primary indicator of the severity of colonic inflammation and cancer [17]. Colonic inflammation involves the disruption of the colonic mucosal architecture and infiltration of inflammatory cells, resulting in the thickening of the lamina propria, which is considered a cause of colon shortening [18]. As shown in Figure 4A,B, the shortening of colon length was observed in HFD+AOM/DSS group, but 5-ASA, CPT, and AG significantly restored the length of the colon. Macroscopic tumors were counted, and the number of tumors (diameter: <1 mm, 1–3 mm, >3 mm) was calculated. HFD-fed CAC induction led to colonic neoplasms, whereas the tumor loads in the 5-ASA, CPT, and AG-treated groups were significantly decreased. Taken together, these results suggested that AG inhibited inflammation-induced colon cancer progression in the HFD-propelled CAC mice model.

### 3.5. AG Alleviated Colonic Disease Progression and Tumorigenesis in HFD-Propelled CAC Mice Model

**Figure 5:**
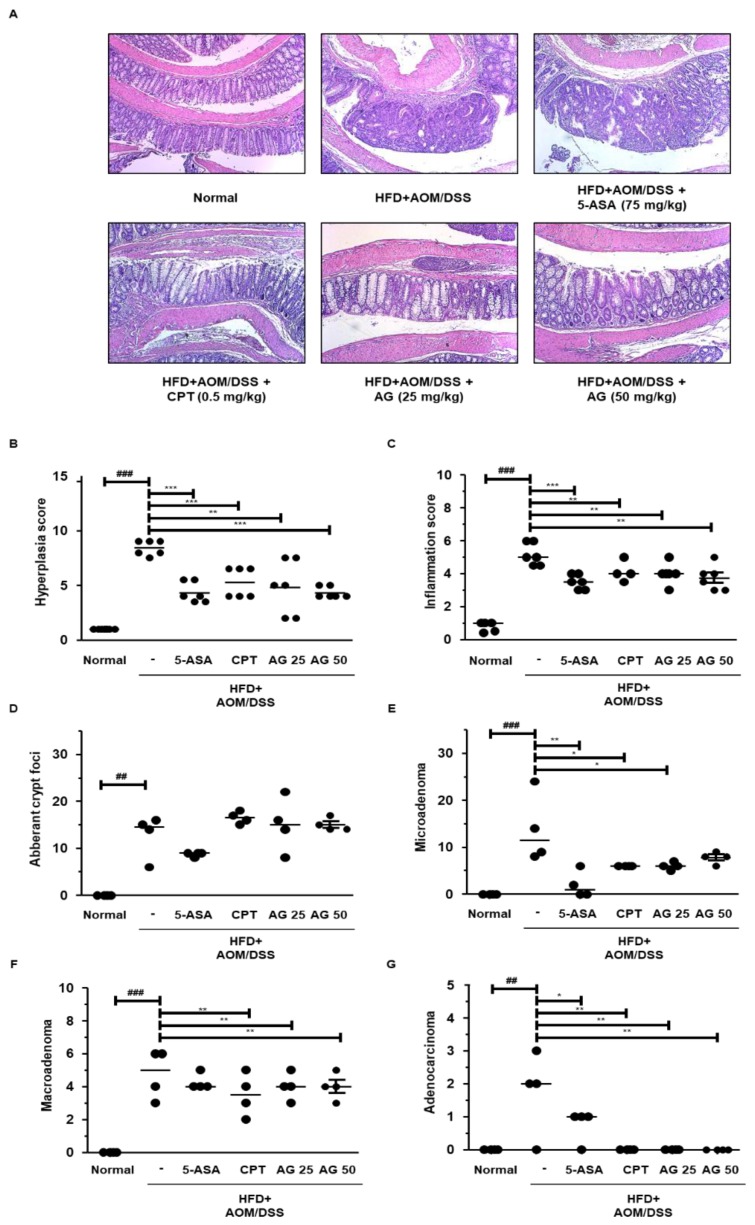
Effects of AG on histological analysis of the colorectal tissues in the HFD-propelled CAC mice model. (**A**) Representative portions of the colon tissues were stained by H&E (original magnification, 100×). (**B**) Hyperplasia score; (**C**) inflammation score; and the degree of (**D**) ACF, (**E**) microadenoma, (**F**) macroadenoma, and (**G**) adenocarcinoma were estimated. The values are the mean ± SD (*n* = 10); ^###^ *p* < 0.001 vs. normal group; * *p* < 0.5, ** *p* < 0.01, *** *p* < 0.001 vs. the HFD+AOM/DSS group; significant differences between the treated groups were determined by ANOVA and Dunnett’s post-hoc test.

HFD increased the susceptibility to DSS-induced inflammation and accelerated the incidence and multiplicity of colorectal tumors in mice. To investigate the effects of AG on colonic crypt morphology in the HFD-propelled CAC mice model, we performed H&E staining and demonstrated the results. As shown in Figure 5A–C, the HFD+AOM/DSS group showed severe inflammation with ulceration, hyperchromatic nuclei, and dysplastic epithelium. However, 5-ASA, CPT, and AG significantly mitigated these symptoms in the colon tissues of HFD-propelled CAC mice. After the development of colonic neoplasms, the HFD+AOM/DSS group mice showed 48 neoplasm-associated lesions as compared to that in the normal group, whereas the neoplasm-associated lesions in the 5-ASA, CPT, AG25, and AG50 administrated groups were 28, 34, 32, and 27, respectively. The histological measurement of tumorigenicity is depicted in Figure 5D–G. In the HFD+AOM/DSS mice group, 35.90% of the lesions were aberrant crypt foci, 23.08% were microadenomas, 33.33% were macroadenoma, and 35.9% were adenocarcinoma. However, 5-ASA, CPT, and AG significantly inhibited tumor progression. Notably, in the high dose AG group, the inhibitory effect of RA was superior to that in the positive control group.

### 3.6. AG Inhibited STAT3 Activation and Expressions of STAT3-Related Proteins in HFD Propelled CAC Mice Model

The STAT3 signaling pathway is involved in the initiation, development, and progression of carcinogenesis in CRC [13]. In present study, we checked that there is significant increase of p-STAT3 manifestation in HFD+AOM/DSS group in contrast with the HFD group, which raises the potential role of STAT3 in HFD propelled AOM/DSS mice model (Supplementary Figure S1). 

**Figure 6:**
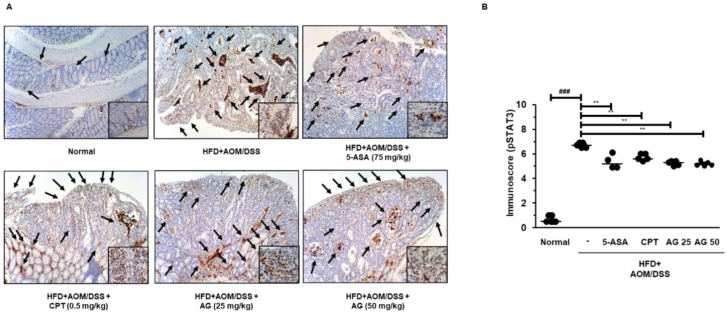
Effects of AG on the activation of STAT3 in the HFD-propelled CAC mice model. (**A**) The manifestation and translocation to the nucleus of pSTAT3 (Tyr705) in the colon tissues were performed by immunohistochemistry. (**B**) pSTAT3 (Tyr705) immune score in each group was estimated. The values are the mean ± SD (*n* = 10); ^###^ *p* < 0.001 vs. normal group; ** *p* < 0.01, *** *p* < 0.001 vs. the HFD+AOM/DSS group; significant differences between the treated groups were determined by ANOVA and Dunnett’s post-hoc test.

**Figure 7:**
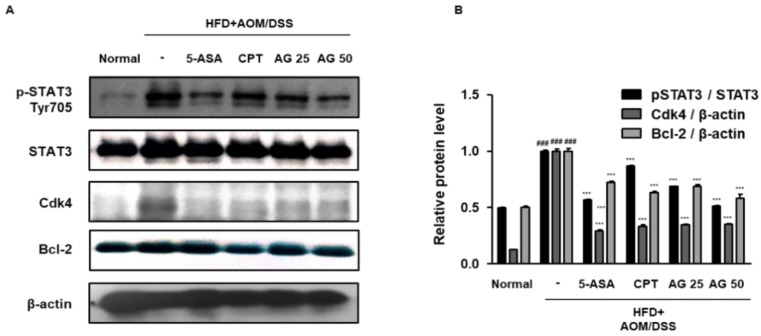
Effects of AG on STAT3 and STAT3 target protein expression in the HFD-propelled CAC mice model. (**A**) The protein extracts were prepared from the colon tissues and western blot analysis was performed for the detection of pSTAT3 (Tyr705), Cdk4, and Bcl-2 protein expression using specific antibodies. The relative protein level was determined by densitometric analysis normalized to the internal controls. The values are the mean ± SD (*n* = 10); ^###^ *p* < 0.001 vs. normal group; *** *p* < 0.001 vs. the HFD+AOM/DSS group; significant differences between the treated groups were determined by ANOVA and Dunnett’s post-hoc test.

To investigate the molecular mechanisms of AG, we evaluated its effect on STAT3 activation in HFD-propelled CAC mice. As shown in Figure 6, the HFD+AOM/DSS mice group demonstrated a marked expression of pSTAT3 as compared to that in the normal group. However, administration of 5-ASA, CPT, AG25, and AG50 inhibited the protein expression of pSTAT3 and its translocation to the nucleus in the tumor tissues of the HFD-propelled CAC mice. Consistently, in the Western blot analysis, the HFD+AOM/DSS group exhibited an increased protein expression of pSTAT3, and the administration of 5-ASA, CPT, and AG significantly inhibited pSTAT3 protein expression. Furthermore, we found that HFD-propelled CAC increased the protein expression of STAT3 target genes, including Cdk4 and Bcl-2, whereas AG significantly inhibited these increases in the colon of HFD-propelled CAC mice (Figure 7).

## 4. Discussion

There is a growing body of literature that recognizes the importance of excess fat intake on immune reactions, chronic inflammation, and CRC [19]. Previous research has found that diet-induced obesity accelerates colon tumor formation, suggesting the contributions of inflammation to tumorigenesis in the HFD-enhanced CRC murine model [20]. Although early epidemiological studies have also shed light on the link between HFD and inflammation-induced CRC risk, there have been a few animal experiments that have explored this relationship. 

There are several possible protocols for the establishment of CRC animal models that recapitulate key features of human CRC, each with its unique advantages and limitations. Thus, any specific experimental topic should be studied by selecting the animal model best suited to resolve the particular tasks. The AOM/DSS-induced CAC mice model that utilizes the chemical induction of DNA damage followed by repeated cycles of colitis is a powerful platform to unravel the pathogenesis of tumor progression induced by colonic inflammation. This model recapitulates the key features of human CAC, such as distal tumor location and aberrant crypt foci-adenoma-carcinoma sequence [21,22]. This study was designed to examine the anti-tumorigenic effects of AG in an HFD-propelled CAC murine model. Numerous methods with AOM and DSS have been used, but none of the experiments have been carried out with the HFD-fed AOM/DSS model based on a single injection of AOM and repeated cycle of DSS. Interestingly, we confirmed that HFD exacerbated the incidence and severity of tumorigenesis in the AOM/DSS-induced CAC mice model. The number of neoplasm-associated lesions in the HFD-fed CAC mice group was about two times higher than that of the normal diet-fed CAC mice group. In addition, HFD-fed CAC induction remarkably accelerated the progression of adenomas to adenocarcinomas when compared to that observed in the AOM/DSS group (Figure 2 and Table 3). 

Meanwhile, recent researchers presented a concern with diet used in obesity-related disease models. It has been noted that animal studies with a 60% fat content are potentially not as relevant to human physiology as those which use a 45% HFD [22]. We were also concerned about using HFD, which can more closely mimic what happens in humans, so we used 45% HFD rather than 60% HFD in the present study.

From our study, it appears that AG remarkably lowered the number of cancer deaths and alleviated the disease-related symptoms in HFD-fed CAC mice (Figure 3). Interestingly, we confirmed that HFD significantly decreased body weight and that AG treatment markedly inhibited the loss of body weight. This result parallels the previous observation that HFD rapidly promotes body weight loss during the carcinogenic period [23]. As maintenance of body weight is important during cancer treatment and recovery, these results support that AG ameliorated the pathological symptoms of HFD-propelled CAC mice. 

The HFD+AOM/DSS group mice developed colorectal carcinoma which was promoted by an inflammation phase, as demonstrated by the shortening of colon length and the development of multiple colonic tumors. On the contrary, AG inhibited the shortening of colon and reduced the tumor burden in HFD-propelled CAC mice (Figure 4). These results were in agreement with those of the previous studies, supporting the notion that AG has anti-inflammatory effects on colitis and CAC. The HFD-fed CAC mice tumors typically exhibited adenomatous growth with changes ranging from low-grade dysplasia to high-grade dysplasia (dysplasia aberrant crypt foci-adenoma-carcinoma sequence) [24]. As shown in Figure 5, AG markedly decreased the pathological indications of inflammation and hyperplasia, which was similar to 5-ASA and CPT. Interestingly, AG significantly suppressed aberrant crypt foci-adenoma-adenocarcinoma sequence in the HFD+AOM/DSS group. These results suggest that AG has antitumor effects on HFD-propelled CAC in mice which involves the drastic progression from inflammation to malignancy. 

The progression of aberrant crypt foci (ACF) to polyps, and subsequently, to adenocarcinoma parallels the accumulation of several biochemical alterations [25]. Our previous results showed that the chemopreventive effects on AOM/DSS-induced CAC by AG was accompanied by the modulation of NF-κB-related cellular proliferation [17]. An emerging body of evidence indicates that NF-κB and STAT3 have been identified as active participants in colon cancer progression and the inhibition of the activation of these two proteins has the potential to prevent and treat colon cancer [25]. Many researchers have also used the AOM/DSS model to demonstrate the role of the JAK/STAT pathway [26]. On the contrary, the STAT3 signaling pathway has been shown to link obesity, inflammation, and cancer, suggesting a target for the treatment of obesity-associated pathologies [27,28]. 

In the present study, we checked that significant increase of p-STAT3 manifestation in mice treated with HFD+AOM+DSS in contrast with HFD group where HFD group and HFD+AOM+DSS group were fed same fat level diet. In addition, the HFD+AOM/DSS group showed marked expression of pSTAT3 as compared to that in the AOM/DSS group (Supplementary Figure S1). This finding suggests that HFD plays a key role in AOM/DSS-induced CAC model via STAT3 signaling pathway. Persistent activation of STAT3 and cyclin D1 overexpression also contribute to enhanced cellular proliferation in colorectal tumor growth [27]. In the present study, AG inhibited constitutive activation and nuclear translocation of STAT3 and modulated STAT3 target protein expression (Figure 6 and Figure 7). These observations are supported by a previous study that demonstrates that inhibition of the STAT3 signaling pathway decreased the viability of colon cancer cells due to apoptosis and cell-cycle arrest through the down-regulation of Bcl-2 [28]. 

## 5. Conclusions

To the best of our knowledge, our study provides evidence that HFD exacerbates the development and progression of carcinogenesis in CAC using the AOM/DSS model based on a single injection of AOM and repeated cycle of DSS. Further, as an extension of our previous data that showed that AG suppressed AOM/DSS-induced CAC and modulated the adipogenesis-related signaling pathway in HFD-induced obesity mice, our study demonstrated that AG significantly suppressed the development and progression of HFD-propelled CAC through modulating the STAT3 signaling pathway. Therefore, further studies are warranted to explore the efficacy of other chemopreventive agents against obesity-linked CAC in humans. 

## Figures and Tables

**Table 1:** Scores of histopathological hyperplasia.

Histological Parameters		Description	Score
Mucosa			
Non-dysplastic Epithelium		Mild (less than two-fold) crypt length	1
		Intense crypt length with hyperchromatic CEC	2
Dysplastic Epithelium		Dysplastic epithelial region (region < 20%)	1
		Dysplastic epithelial region (20% < region < 50%)	2
		Dysplastic epithelial region (50% < region < 90%)	4

**Table 2:** Scores of histopathological inflammation.

Histological Parameters	Description	Score
Mucosa		
Epithelial cell	Prolonged epithelial cell or crypt	1
	Destruction of barrier	2
	Ulcer (30% < loss < 60%)	3
	Ulcer (loss > 60%)	4
Immune cell	Infiltration (mild)	1
	Infiltration (moderate)	2
	Infiltration (severe)	3
Submucosa		
Immune cell	Infiltration (mild)	1
	Infiltration (moderate)	2
	Infiltration (severe)	3

**Table 3:** Histological parameters of distal colon for assessment of neoplasm.

Group	ACF (%)	Microadenoma (%)	Low-Grade Macroadenoma (%)	High-Grade Macroadenoma (%)	Adenocarcinoma (%)	Neoplasm-Associated Lesions (N)
Normal	0.00%	0.00%	0.00%	0.00%	0.00%	0
AOM/DSS	33.33%	26.19%	19.05%	7.14%	2.38%	21
HFD	0.00%	0.00%	0.00%	0.00%	0.00%	0
HFD+AOM/DSS	35.00%	22.50%	12.50%	20.00%	5.00%	40

## Supplementary Materials

The following are available online at https://www.mdpi.com/2079-7737/9/2/24/s1, Figure S1: Effects of HFD on the activation of STAT3 in the AOM/DSS-induced CAC mice model. The manifestation of pSTAT3 (Tyr705) in the colon tissues were performed by immunohistochemistry (original magnification, 100×).
