# Supplementary material for: High-Fat Diet Propelled AOM/DSS-Induced Colitis-Associated Colon Cancer Alleviated by Administration of Aster glehni via STAT3 Signaling Pathway
Source: Biology (Basel). 2020 Feb 2;9(2):24. doi: 10.3390/biology9020024 (PMC7168234; doi:10.3390/biology9020024)
Supplement: Supplementary file 1 [file biology-09-00024-s001.pdf]

biology

MDPI

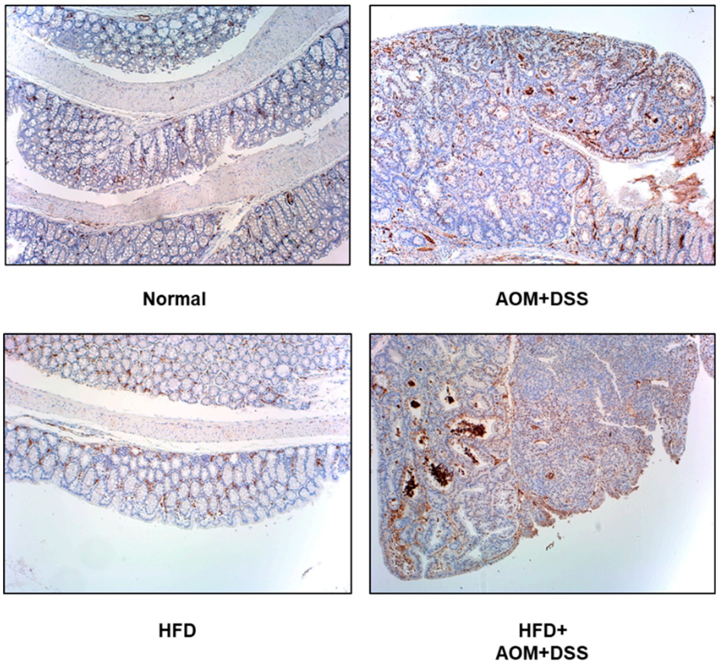

Supplementary Figure S1. Effects of HFD on the activation of STAT3 in the AOM/DSS-induced CAC mice model. The manifestation of pSTAT3 (Tyr705) in the colon tissues were performed by immunohistochemistry (original magnification, 100×).
